# Use and Efficacy of the Lob to Achieve the Offensive Position in Women´s Professional Padel. Analysis of the 2018 WPT Finals

**DOI:** 10.3390/ijerph17114061

**Published:** 2020-06-06

**Authors:** Adrián Escudero-Tena, José Fernández-Cortes, Javier García-Rubio, Sergio J. Ibáñez

**Affiliations:** Training Optimization and Sport Performance Research Group (GOERD), Sport Science Faculty, University of Extremadura, 10005 Caceres, Spain; jfernandxb@alumnos.unex.es (J.F.-C.); jagaru@unex.es (J.G.-R.); sibanez@unex.es (S.J.I.)

**Keywords:** female paddle tennis, tactic, performance, in-game analysis

## Abstract

Studies that analyze the actions carried out by paddle tennis players during the point are scarce. The present investigation characterizes every action in which a stroke by a pair in a defensive position sends the ball over the position of a pair in an offensive position. It is a descriptive and observational study of quantitative methodology. The sample consisted of 1324 actions, statistical analysis units, from the women’s circuit in the 2018 World Padel Tour (WPT) season. For this study, various situational, dependent, and independent variables were analyzed. The results showed the number of times the categories of each variable occurred, as well as the significant relationships between the independent variable *kind of hit* and the dependent variables “actions that facilitate the possible change of position” (AFPCP) and “incidences in the game” (IG). The conclusion is that the lob is the most effective kind of hit (*CSR* = 4.9) to achieve the offensive position (*CSR* = 11.4), even if the point does not finish (*CSR* = 5.8), leading to more position exchanges during the same point in the AFPCP. These findings are of great interest since they give information about how and why certain behaviors produce a certain result.

## 1. Introduction

Padel (also known as paddle tennis) is one of the most commonly practiced racket sports in Spain, as the data shows an estimate of 4.2 million padel practitioners in 2015 [1]. Likewise, this growth is evident from the perspective of federated padel where the number of licenses increased spectacularly from 1995 (3075) to 2018 (72,266). On the contrary, the number of licenses in other racket sports such as tennis, badminton, squash, or table tennis have decreased or remained stable [2]. Despite the growing popularity of this sport, various studies indicate that the main racket sports are badminton, squash, table tennis, and tennis [3,4]. As a consequence, research on padel practitioners is quite limited compared with tennis [5,6,7], badminton [8,9,10], and squash [11,12]. In turn, different reviews carried out on padel [13,14,15] have indicated that although padel research has increased in recent years, there are still few studies providing scientific knowledge about this sport modality.

Specifically, there is interest in the description of competition and the discovery of performance indicators through game analysis [16,17,18,19,20]. This kind of analysis allows data extraction from spontaneous behaviors and in real contexts of competition, providing objective information about real game situations which is of great interest for the development of competition strategies, the design of training tasks, applying feedback on certain behaviors, or improving athletes´ decision making [21,22]. One of the most investigated factors in the studies are the performance indicators that increase the effectiveness of scoring. These studies have highlighted the relationship between earning points and occupying offensive positions or others near to the net [23,24,25]. Thus, the objective of the pair who are in the defensive zone is to fight to achieve the offensive position and that of the players who are in the offensive zone is to fight to keep it [26,27].

However, studies that analyze the game process are lacking, more specifically, the actions that padel players perform during the point, with the objective of detecting those technical and tactical behaviors that are most effective both to maintain and to recover the net or the offensive position [28]. With the objective of being able to access offensive positions from the back of the court, players can make different kinds of hits, among which the lob is the most common technical action performed by professional men padel players [29,30]. Although, no studies have been found that analyze the different actions that women´s professional padel players perform to approach the net.

Research on sports performance analysis has shown different responses in men and women athletes during competition [19,25,31]. In padel, an aspect studied in terms of gender refers to the analysis of the consequences of the game in padel. Thus, the temporal aspects of the game have been investigated, finding that women professional players seem to obtain significantly higher values in total playing time [32,33,34]. Moreover, the actual playing time has been assessed at approximately 30% and 35% of the total time [25,32,34,35] and therefore less than the rest time [20], is significantly higher for women. Another aspect analyzed in terms of gender in padel corresponds to the duration of the points and the rest between points, finding that the average duration of the points varies between 10–15 s [32,33,34], being significantly longer in women [32,33]. Furthermore, studies have quantified the number of hits in professional players and have shown an average of between 8 and 10 hits per point [25,32,35], this number is significantly higher in the women’s category [32]. Regarding the actions in padel, the backhand volley is the most commonly used hit by men padel players, while the bandeja [tray shot] is the most used gesture by women paddle players [19]. Thus, the results of these investigations are an example of the differences that exist in the analysis of the consequences of the padel game according to the gender of the athletes.

An analysis of the literature revealed the scarcity of work on women´s padel and the non-existence of studies that refer to the investigation of the game process. More specifically, about the actions that women padel players perform during a point to achieve the offensive position. Therefore, the intention is to respond to this absence by investigating the unit of analysis: a hit from the pair in the defensive position that goes past the position of the pair in the offensive position and subsequent actions. For this unit of analysis to occur, two situations must coincide. On the one hand, one pair must occupy the defensive position while the other pair occupies the offensive position and, moreover, the pair in the defensive position must make a hit that goes past the pair occupying the offensive position. Thus, the objectives of the research were two: (i) to characterize any action in which there was a hit by the pair in a defensive position that went past the position of the pair in an offensive position; (ii) to analyze the relationships between the kind of hit and the actions that facilitate the possible change of position and incidences in the game.

## 2. Materials and Methods

### 2.1. Research Design

According to Montero and León, the research methodology is quantitative and more specifically descriptive using an arbitrary code of natural observation [36]. Furthermore, according to Ato, López, and Benavente [37] this study was empirical, ideographic, multidimensional, and longitudinal.

### 2.2. Participants and Sample

The study participants were the top ten padel players who competed on the WPT women’s circuit in the 2018 season. The 14 finals of the tournaments of the 2018 WPT women’s circuit were analyzed, identifying 324 moves (statistical analysis units), where there was a hit with which the pair in the defensive position passed the pair in the offensive position.

### 2.3. Study Variables

The situational variables were *kind of tournament* based on the pairs playing each tournament and the number of points at stake. *The pair in the defensive position* and *the pair in the offensive position* at the start of the analyzed unit. *Partial result in the game or tie break* and *partial result in the set* of the pair initially in a defensive position in the analyzed move. Difference of points and difference of games of the pair in the defensive position in the action. *Key point*, points that could have an impact on the result, in which either pair had the option of getting a game, set, or match. Set, game in the set, or tie break to which the analyzed action and streak of the pair in a defensive position initially when the unit of analysis occurred, belonged.

The independent variable was the *kind of hit* or hit with which the pair in a defensive position passed the pair in an offensive position, with their categories being lob and not lob.

The dependent variables were *effectiveness of the kind* of hit with its valid (play would continue) or invalid (play would end) range. *Achieving the offensive position*, making a hit that passed the pair in the offensive position and was valid; this variable concerns whether or not this hit served to achieve the offensive position. *Effectiveness of achieving the offensive position*, once the pair in the defensive position has achieved the offensive position, this distinguishes between whether the point ended in their favor, against them, if it did not end and the game continued or if they did not achieve the offensive position because the kind of hit was invalid. *Consequence of achieving the offensive position* is categorized into if the point did not end or if the point ended with a winner, a forced error, or an unforced error, or if they did not achieve the offensive position because the kind of hit was invalid. The *rally* was the number of hits during a point and its range has been inductively constituted, as there were no records to perform a cluster analysis. The *rally order* was the hit from the pair in the defensive position that went past the pair in the offensive position and the categories have been inductively constituted. The *hits after achieving the offensive position*, this variable refers to the number of hits that occurred from when the pair that was in a defensive position initially achieved the offensive position until a winner, an unforced error or a forced error occurred; with categories none, one or two hits, three, four or five hits, six hits or more. In addition, it may be that this pair did not achieve the offensive position because the kind of hit was invalid, or that the point did not end. The last independent variable was *the final position of the pair initially in the offensive position*.

It should be emphasized that the variables *kind of hit*, *effectiveness of the kind of hit*, *achieving the offensive position*, *effectiveness of achieving the offensive position* and *consequence of achieving the offensive position* belong to the group of variables called “actions that facilitate the possible change of position” (AFPCP). While the variables *rally*, *rally order*, *hits after attaining the offensive position* and *the final position of the pair initially in the offensive position* belong to the group of variables called “incidences in the game” (IG).

### 2.4. Instruments and Materials

The instruments and materials used in the research were an “ad hoc” observation sheet, tailored for the present study as recommended by Anguera and Hernández-Mendo [38], which was used to build a system of categories adjusted to the unit of analysis. In addition, LINCE software, version 1.4 [39] was used to record the AFPCP and IG that occurred during the unit of analysis.

### 2.5. Process

A bibliographic review was carried out, the problem approach was devised and an "ad hoc" observation sheet was constructed. Once the variables and categories had been defined, the LINCE software [39] was used for recording the data. So, the observed category was recorded after each move in which the unit of analysis occurred. The only observer, graduated in sports sciences, with extensive experience in padel and trained to perform the recordings, analyzed the entire sample twice in order to calculate intra-observer reliability through *Cohen’s Kappa* (*K*) coefficient [40], to ensure the consistency of the data, exceeding the Igartua [41] recommendation to analyze between 10% and 20% of the analysis units. The average reliability obtained through the *Kappa* coefficient was 0.985, so it can be affirmed that there is agreement in the analyzed intra-observer records. Following Landis and Koch [42], the record obtained an “almost perfect” degree of agreement between 0.81–1.00, as is demonstrated in the *Kappa* values [40].

### 2.6. Statistical Analysis

A descriptive analysis was performed to obtain the frequency and percentage of times that the categories of the variables were produced. An inferential analysis was conducted of the associations among the variables, using contingency tables, including the *Chi-squared* (*χ*^2^) test. The association strength among the variables was calculated, using *Cramer´s V* (*Vc*) coefficient [43]. This association was interpreted following the recommendations of Crewson [44]. The contingency tables identified the associations among the categories of the variables with *corrected standardized residuals* (*CSR*), with values greater than | 1.96 | [45]. Statistical analyses were performed using SPSS v.21 software (IBM Corp. Released 2012. IBM SPSS Statistics for Windows, NY, USA). Statistical significance was set at *p* < 0.05.

## 3. Results

This section presents the results of the analyses. Table 1 shows the descriptive results of the situational variables of the investigation.

The tournament with the most analyzed action was the open (*73.6%*) and pair 1 the players who made the most plays both in a defensive position (*43.9%*) and an offensive position (*43.9%*). All the percentages in the partial result of the game or tie break were around more than thirty percent. In the difference in points during games, the category that recorded the highest value was the difference of one point (*45.0%*). In the partial result of the set the category that recorded the highest value was winning (*36.3%*). In the difference of games during the disputed sets, the category that had the highest number was the difference of one game (*38.8%*). Most points were not key (*69.4%*), more moves were played during the second set (*49.7%*) and during the first four games of each set (*44.5%*). Finally, the studied moves occurred to a greater extent when the players had won the previous point (*25.0%*) and when they had lost it (*25.2%*).

Table 2 presents the descriptive analysis of the dependent and independent variables of the study.

The lob is the most used hit (*85.4%*), the hits are usually valid (*79.5%*), and, in most cases, pairs achieve the offensive position (*70.4%*). It is not usually possible to finish the point (*36.9%*), the rallies are very short (*33.2%*) or short (*33.5%*) and the number of the hit in which the pair in the defensive position makes a hit to go past the pair in the offensive position is between the second and sixth hit, that is to say, very soon (*53.6%*). Finally, the pair initially in an offensive position returns to the same position on many occasions (*50.3%*).

Table 3 shows the results of the relationships between the kind of hit variable with the rest of the dependent variables.

The results indicate significant associations, either low or moderate, in all the variables except for the rally order. On the other hand, the effectiveness of the kind of hit is valid (*CRS* = 4.9) and the pair achieved the offensive position (*CRS* = 11.4) when the hit was a lob. The effectiveness and consequence of achieving the offensive position is that the point does not end, followed by other categories. The IG indicates that when the hit is a lob the rally is normal (*CRS* = 3.2) and the final position of the pair in the offensive position is the defensive position (*CRS* = 7.3).

## 4. Discussion

The objective was to characterize any move in which there was a hit by the pair in a defensive position that destabilized the position of the pair in an offensive position and subsequent actions. Although there are several investigations that have analyzed temporal aspects, number, and kind of hitting or performance indicators in women’s padel [16,17,18,19,20], no studies have been found that analyze the different actions that padel players perform during the point to get the offensive position. The results show that the women’s finals of the WPT 2018 season were extremely close, as demonstrated by the variable points difference, game difference, and streak. This equality is understandable since only the fittest pairs in the WPT women’s circuit get to the finals of the tournaments and have won all the matches of the tournament previously played (semifinals, quarterfinals, fourth round, etc.). Thus, it is essential for paddle players to train and get used to playing disputed matches, involving great fatigue and in conditions of extreme equality.

The results of the present investigation indicate that the lob is the *kind of hit* most used in “actions that facilitate the possible change of position” and *the effectiveness of the kind of hit* is valid. Torres-Luque and collaborators [25] affirm that the backhand volley is the most commonly used kind of hit in the men’s category, while the lob and the shot appear more frequently in the women’s category. Professional players use the lob a great deal, mostly from the back of the court [19,46,47]. For their part, García-Benítez et al. [32], highlight in their study that women execute a large number of lobs, representing 32.1% of the hits in a match except for serves. Although these investigations have analyzed the consequences of the game and not the game process, it is expected that the padel players use the lob from the defensive position to alleviate the pressure from the players in attack and then to try to take the offensive position from them. Thus, it is necessary for women padel players to introduce this hit, the lob, as a fundamental element in their training, since on many occasions the lob is treated as a basic hit and therefore is not given much of importance.

Pairs in a defensive position initially *achieve the offensive position*. However, the *effectiveness of getting the offensive position* or *the consequence of getting the offensive position* is that they do not finish the points. Therefore, not finishing the point leads to its continuity and not to its end in favor or against. These results do not coincide with those set forth in the investigations of various authors [23,24,25], who conclude that winning points and occupying the offensive position is related, perhaps because their work only collects information once the point has ended and not during it. In a padel match, pairs can exchange their positions, from defensive to offensive or vice versa, on several occasions during the same point until the last hit. Therefore, padel players must train to have a good physical condition in such a way as to allow them to get quickly from one position to another during all the games they play.

The incidence of this kind of hitting in the game shows a reduction in the duration of the rally, making them short or very short sequences. These results are in line with those reported in several studies [25,32,34,46], where an average of between 8 and 10 hits per point was found in high-level padel. Professional padel players try to get the initiative with the aim of winning the point with each hit. So, they must execute each hit with an intention or purpose in such a way that it benefits them in the game.

On the other hand, when the kind of hit is a lob, the effectiveness is valid and the pair in the defensive position initially gets the offensive position. However, the effectiveness of achieving the offensive position is that the point does not end on more occasions than ending in favor or against in the AFPCP. These results are in line with what several authors showed in their research [29,30], whereas in our work, the process of the game in padel was studied. Thus, their conclusions indicate that in men padel players can make different kinds of hits with the aim of being able to access offensive positions from the back of the court, presenting the lob as the most-used technical action. They found in their study the probability of continuing the point after using the lob, as a prior action to attaining the net, was significantly higher compared to the use of actions at medium or low altitude [29,30]. Therefore, in unfavorable situations, padel players must make a lob, with the aim of unbalancing the attack of their opponents and later snatching the offensive position thus achieving the initiative in the point. In addition, once they achieve the net, they must be able to win the point in that position and avoid its continuity, as well as more possible exchanges of position, so they must train this situation.

Finally, the rally is normal (from 17 to 24 hits) in the IG when the kind of hit is a lob, so it is verified that when this kind of hit takes place, the probability of continuing the point increases, as indicated in other studies [29,30]. Therefore, padel players must be prepared to physically and mentally endure rallies of more hits than usual.

## 5. Conclusions

The objectives of the study having been achieved, the conclusions can be drawn that the lob is the most commonly used and valid hit by the women´s pairs in the defensive position to pass the position of the pairs in the offensive position. However, the point does not end on most occasions, thus allowing continuity in the game and leading to more position exchanges between pairs during the same point, followed by ending the point in favor, and in fewer cases, against.

Padel is a sport that is booming at a global level, both in amateurs and professionals, being a sport that encourages diversity (it can be played by men, women, children, or the elderly). For all these reasons, and due to the lack of literature, this information is very valuable and of great use to future researchers who contemplate the possibility of carrying out this kind of study.

Moreover, although this work is scientific, it provides very novel theoretical results that can be used by any padel player both in a match and in training, in addition to being an aspect to be taken into account by coaches when developing and planning their sessions. This study provides information about a padel hit that occurs on many occasions and that allows the initiative to be achieved in the point, so these results help improve the athlete’s decision making, the development of competitive strategies, or the design of specific paddle tennis tasks. It helps to ascertain the effects of the variable situations (partial result of the game or tie break, set, kind of tournament etc.). On the athlete’s performance. It analyzes the technical-tactical behavior of athletes based on the extraction of actions in a real game environment. It provides information about the physical performance of the athlete through the analysis of their movements or actions during the real context of the game. In addition, it provides information about “why” and “how” a certain action produces a specific result; information that is lacking in padel today. Finally, it would be interesting for future research to carry out this same analysis with a larger sample, in men players, and in different categories.

## Figures and Tables

**Table 1:** Descriptive results of the situational variables.

Situational Variables	Categories	*n*	*%*
*Tournament kind*	Open	975	73.6
	Master	277	20.9
	Master Final	72	5.4
*Pair in defensive position*	Pair 1	581	43.9
	Pair 2	315	23.8
	Pair 3	337	25.5
	Pair 4	52	3.9
	Pair 5	39	2.9
*Pair in offensive position*	Pair 1	607	45.8
	Pair 2	310	23.4
	Pair 3	330	24.9
	Pair 4	42	3.2
	Pair 5	35	2.6
*Partial result of the game or tie break*	Tying	454	34.3
	Winning	411	31.0
	Losing	459	34.7
*Difference in points*	Tying	455	34.4
	Difference of one point	596	45.0
	Difference of two points	213	16.1
	Difference of three points or more	60	4.5
*Partial result of the set*	Tying	450	34.0
	Winning	480	36.3
	Losing	394	29.8
*Difference in games*	Tying	452	34.1
	Difference of one game	514	38.8
	Difference of two games	226	17.1
	Difference of three games or more	132	10.0
*Key point*	Yes	405	30.6
	No	919	69.4
*Set*	First	504	38.1
	Second	526	49.7
	Third	294	22.2
*Game on set or tie break*	Of the first to the fourth game	589	44.5
	Of the fifth to the eighth game	497	37.5
	Of the ninth to the twelfth game	193	14.6
	Tie break	45	3.4
*Streak*	Won the previous point	331	25.0
	Won the previous two points	175	13.2
	Won the previous three points or more	175	13.2
	Lost the previous point	333	25.2
	Lost the previous two points	174	13.1
	Lost the previous three points or more	130	9.8
	First point of the match	6	0.5

**Table 2:** Descriptive results of the dependent and independent variables.

Dependent and Independent Variables		Categories	*n*	*%*
Actions that facilitate the possible change of position	*Kind of hit*	Lob	1131	85.4
		Not lob	193	14.6
	*Effectiveness of the kind of hit*	Valid	1052	79.5
		Not valid	272	20.5
	*Achieving the offensive position*	Yes	932	70.4
		No	392	29.6
	*Effectiveness of achieving the offensive position*	The point ends in favor	271	20.5
		The point ends against	167	12.6
		The point does not end	488	36.9
		The offensive position is not achieved	398	30.1
	*Consequence of achieving the offensive position*	Unforced error	162	12.2
		Forced error	107	8.1
		Winner	170	12.8
		The point does not end	489	36.9
		The offensive position is not achieved	396	29.9
Incidences of the game	*Rally*	Very short (2 to 8 hits)	440	33.2
		Short (9 to 16 hits)	443	33.5
		Normal (17 to 24 hits)	221	16.7
		Long (25 to 32 hits)	129	9.7
		Very long (33 to more hits)	91	6.9
	*Rally order*	Very soon (from 2nd to 6th hit)	708	53.5
		Soon (from 7th to 11th hit)	313	23.6
		Normal (from 12th to 16th hit)	142	10.7
		Late (from 17th to 21st hit)	81	6.1
		Very late (from 22nd to more hits)	80	6.0
	*Hits after attaining the offensive position*	None, one or two hits	183	13.8
		Three, four or five hits	116	8.8
		Six hits or more	139	10.5
		The point does not end	490	37.0
		The offensive position is not achieved	396	29.9
	*The final position of the pair in the offensive position*	Defensive position	464	35.0
		Transition	95	7.2
		Offensive position	666	50.3
		Staggered	99	7.5

**Table 3:** Analysis of the kind of hit with respect to the dependent variables.

Dependent Variables		*χ* ^2^	*gl*	*p*-Value	*Vc*	*p*-Value	Association Strength	Categories	Lob	N-lob
									*CSR*	*CSR*
Actions that facilitate the possible change of position	*Effectiveness of the kind of hit*	23.880	1	0.000	0.134	0.000	Low	Valid	4.9	−4.9
								Not valid	−4.9	4.9
	*Achieving the offensive position*	130.091	1	0.000	0.313	0.000	Moderate	Yes	11.4	−11.4
								No	−11.4	11.4
	*Effectiveness of achieving the offensive position*	131.891	3	0.000	0.316	0.000	Moderate	The point ends in favor	2.6	−2.6
								The point ends against	4.1	−4.1
								The point does not end	5.8	−5.8
								The offensive position is not achieved	−11.4	11.4
	*Consequence of achieving the offensive position*	132.134	4	0.000	0.316	0.000	Moderate	Unforced error	3.5	−3.5
								Forced error	1.3	−1.3
								Winner	2.7	−2.7
								The point does not end	5.9	−5.9
								The offensive position is not achieved	−11.4	11.4
Incidences in the game	*Rally*	30.813	4	0.000	0.153	0.000	Low	Very short (2 to 8 hits)	−5.3	5.3
								Short (9 to 16 hits)	1.9	−1.9
								Normal (17 to 24 hits)	3.2	−3.2
								Long (25 to 32 hits)	1.3	−1.3
								Very long (33 to more hits)	0.1	−0.1
	*Rally order*	4.400	4	0.355	0.058	0.355	Little	Very soon (from 2nd to 6th hit)	−0.1	0.1
								Soon (from 7th to 11th hit)	-0.3	0.3
								Normal (from 12th to 16th hit)	0.4	-0.4
								Late (from 17th to 21st hit)	1.6	−1.6
								Very late (from 22nd to more hits)	−1.4	1.4
Incidences in the game	*Hits after attaining the offensive position*	128.951	4	0.000	0.312	0.000	Moderate	None, one or two hits	2.0	−2.0
								Three, four or five hits	3.3	−3.3
								Six hits or more	2.9	−2.9
								The point does not end	5.6	−5.6
								The offensive position is not achieved	−11.3	11.3
	*The final position of the pair in the offensive position*	113.586	3	0.000	0.293	0.000	Low	Defensive position	7.3	−7.3
								Transition	−9.1	9.1
								Offensive position	−1.9	1.9
								Staggered	−0.8	0.8

*χ*^2^ (*Chi-squared*); *gl* (*degrees of freedom*); *Vc* (*Cramer´s V*); *CRS* (*corrected standardized residuals*).
